# Filtered aquatic small tubular mesh-bottomed containers (FAST-MC): A low-cost, efficient method for rearing zebrafish larvae in filtered water

**DOI:** 10.1016/j.mex.2025.103453

**Published:** 2025-06-21

**Authors:** M. Caballero, S. Robles, VP Connaughton

**Affiliations:** Department of Biology, Center for Neuroscience and Behavior, American University, WA, DC 20016, USA

**Keywords:** *Danio rerio*, Larval ecology, Aquaculture, FAST-MC: a novel, low-cost, efficient method for rearing zebrafish larvae

## Abstract

Care for larval zebrafish (*Danio rerio*) can be taxing and time-intensive, as embryos/larvae are housed in petri dishes or well plates which require daily care. Lack of filtration in the dishes can affect overall water quality, especially in ecotoxicological exposures which can last weeks. This report describes an alternative method, Filtered Aquatic Small Tubular Mesh-bottomed Containers (FAST-MC), which separates larvae into petri dish-sized housing containers maintained in a larger volume of filtered water, mimicking the larger, recirculating systems used for adults. To validate our method, we raised zebrafish larvae in FAST-MC containers for 4-weeks and compared overall survival, behavior, and water quality with larvae reared for 4-weeks in standard deep dish petri dishes. Overall, this method provides a low-cost alternative for housing larval zebrafish that is amenable to experimental manipulation and suitable for toxicological and/or pharmacological studies.

• An easily constructed float can hold small mesh-bottomed (hatchery-type) containers in a larger aquarium allowing constant water filtration and improved water quality.
• Each mesh bottom container can serve as a housing container for experimental exposures, similar to multiwell plates.
• Zebrafish larvae raised in the mesh bottom containers had increased survival and activity compared to larvae raised in standard petri dishes.

## Graphical abstract

**Figure:**
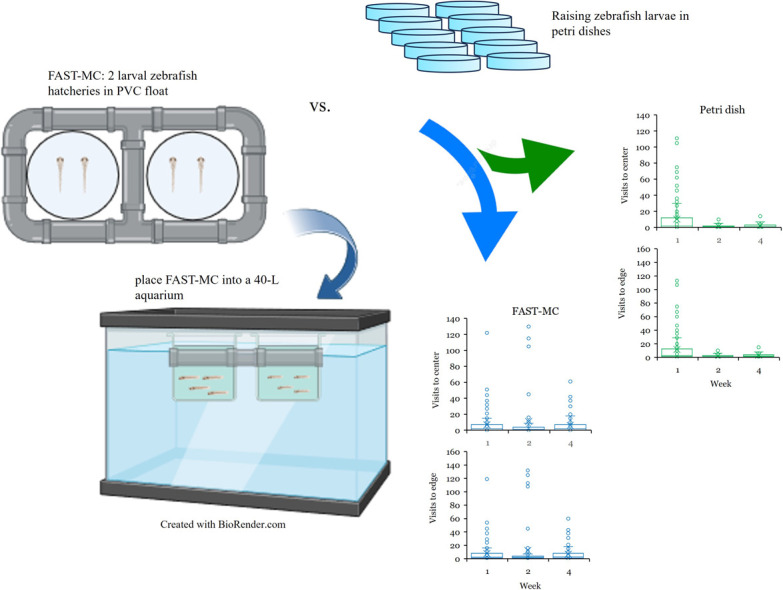

Specifications table

**Table:** 

Subject area:	Agricultural and Biological Sciences
More specific subject area:	Larval fish care and maintenance
Name of your method:	FAST-MC: a novel, low-cost, efficient method for rearing zebrafish larvae
Name and reference of original method:	P Cattin, P Crosier. A nursery that improves zebrafish fry survival. Methods Cell Biol. 77 (2004) 593–598. A Norton A, K Franse, T Daw, L Gordon, P Vitiello, M Kinkel. Larval rearing methods for small-scale production of healthy zebrafish. Eastern Biologist. 2019 (2019) 33–46. S Poureetezadi, E Donahue, R Wingert. A manual small molecule screen approaching high-throughput using zebrafish embryos. Journal of Visualized Experiments. 93 (2014) e52063.
	C Roper, S Simonich, R Tanguay. Development of a high-throughput in vivo screening platform for particulate matter exposures. Environmental Pollution. 235 (2018) 993–1005. W Spomer, A Pfriem, R Alshut, S Just, C Pylatiuk. High-throughput screening of zebrafish embryos using automated heart detection and imaging. Journal of Laboratory Automation. 17 (2012) 435–442. M Westerfield. The zebrafish book: A guide for the laboratory use of zebrafish. (2000) Http://zfin.org/zf_info/zfbook/zfbk.html.
Resource availability:	Supply List: 1. Aqueon Size 10 Glass aquarium (Petco) 2. Aqueon T8 Fluorescent Deluxe Full Hoods, 20″ (Petco) 3. X-acto knife (Ace Hardware) 4. SunGrow Aquarium Tank Water Temperature Digital Thermometer Gauge (Chewy) 5. Fluval® M-Series Submersible Heater (Petco) 6. Extra Large Binder clips, 4 (Amazon) 7. 2 tubing clips (Amazon) 8. Aqueon® QuietFlow Aquarium Power Filter 10 (Petco) 9. Aqueon® Replacement Aquarium Filter Cartridge, Medium (Petco) 10. Aquarium-safe mesh (for filter intake) (Amazon) 11. 4-inch zip ties (Ace Hardware) 12. Fiskars 94,817,797 Micro-Tip Scissors, 5 Inch, Orange (Amazon) 13. 2 black rubber rings (Pentair) 14. Hatchery (2 clear cylinders, 3.5-inch diameter) (Pentair) 15. Milwaukee 12″ Straight Jaw Pliers (Ace Hardware) 16. Husky 1-¼ inch Ratcheting PVC Cutter (Home Depot) 17. Stanley FatMax Measuring Tape (Home Depot) 18. API Freshwater Master Test Kit (Petco) 19. Traceable Conductivity/TDS Pocket Tester with Calibration (Cole-Parmer) 20. Black Sharpie marker (Amazon) 21. VPC 3/4 in x 24 in PVC Sch. 40 Pipe (Home Depot) 22. 4 Charlotte Pipe 3/4 in PVC Schedule. 40 90° S x S Elbow Fitting (Home Depot) 23. 2 Charlotte Pipe 3/4-in x 3/4-in dia S x S x S Tee PVC Fitting (Home Depot)

## Background

*Danio rerio*, zebrafish, are a well-established model organism for developmental biology, molecular biology, genetics, toxicology, and neuroscience. Their applicability is both biological and practical. Zebrafish share developmental characteristics with higher vertebrates including humans, gene expression is highly conserved, and behaviors are easily measured [1]. Zebrafish also spawn year-round, producing large numbers of externally developing eggs that are amenable to experimental observation and manipulation. The use of the zebrafish model to human diseases, in particular [[2], [3], [4]] underscores their translational importance.

Housing conditions can greatly affect zebrafish during development, including type of tank, type of food, stocking density, and lighting regime [[5], [6], [7], [8], [9], [10]]. Husbandry for zebrafish larvae can be labor intensive because larvae typically are housed in multi-well plates or petri dishes [[11], [12], [13], [14], [15]] that must be checked daily. Toxicological studies, which use well plates and petri dishes as the exposure containers, can also be labor intensive for the same reasons. While these containers are efficient, they are also small in volume and limited in depth [14], which allows researchers to use fewer reagents, but can also potentially impact water quality around the larvae [16].

Here we describe a technique that is an alternative to conventional larval rearing methods, can streamline larval care, and can be of use to experimenters performing toxicological studies, particularly for studies that involve long term (several week) exposures. The method, called FAST-MC (Filtered Aquatic Small Tubular Mesh-bottomed Containers), utilizes the small mesh-bottomed hatchery containers found with most zebrafish recirculating systems. These containers fit into a homemade PVC float that can be customized and used in a larger aquarium, providing water filtration (similar to [17]). In this way, each aquarium becomes a separate group, with separate FAST-MC containers housing eggs and/or larvae. Thus, the FAST-MC containers are similar to traditional petri dishes or well plates, with the addition of water filtration. FAST-MC containers also have a larger volume and depth, so daily animal care can be completed more quickly, and larvae are easily visualized, providing efficient, low-cost housing for larvae. To validate our method, we compare water quality, survival, and fish behavior of larvae housed for 4-weeks in petri dishes vs 4-weeks in FAST-MC containers. Our results found that, while general behaviors were comparable between fish raised in the two containers, water quality was better, activity was increased, and survival was higher for larvae housed in FAST-MC containers.

## Method details

### FAST-MC design and set-up

*Aquaria:* 40-L aquaria were filled with system water, which was the same water used in adult stock tanks. Each aquarium was outfitted with a top filter, with the intake covered with black mesh to prevent large debris from clogging the filter. Each aquarium was also fitted with a submersible heater, to maintain temperature at 28–29 °C for the duration of the experiment.

**Fig. 1:**
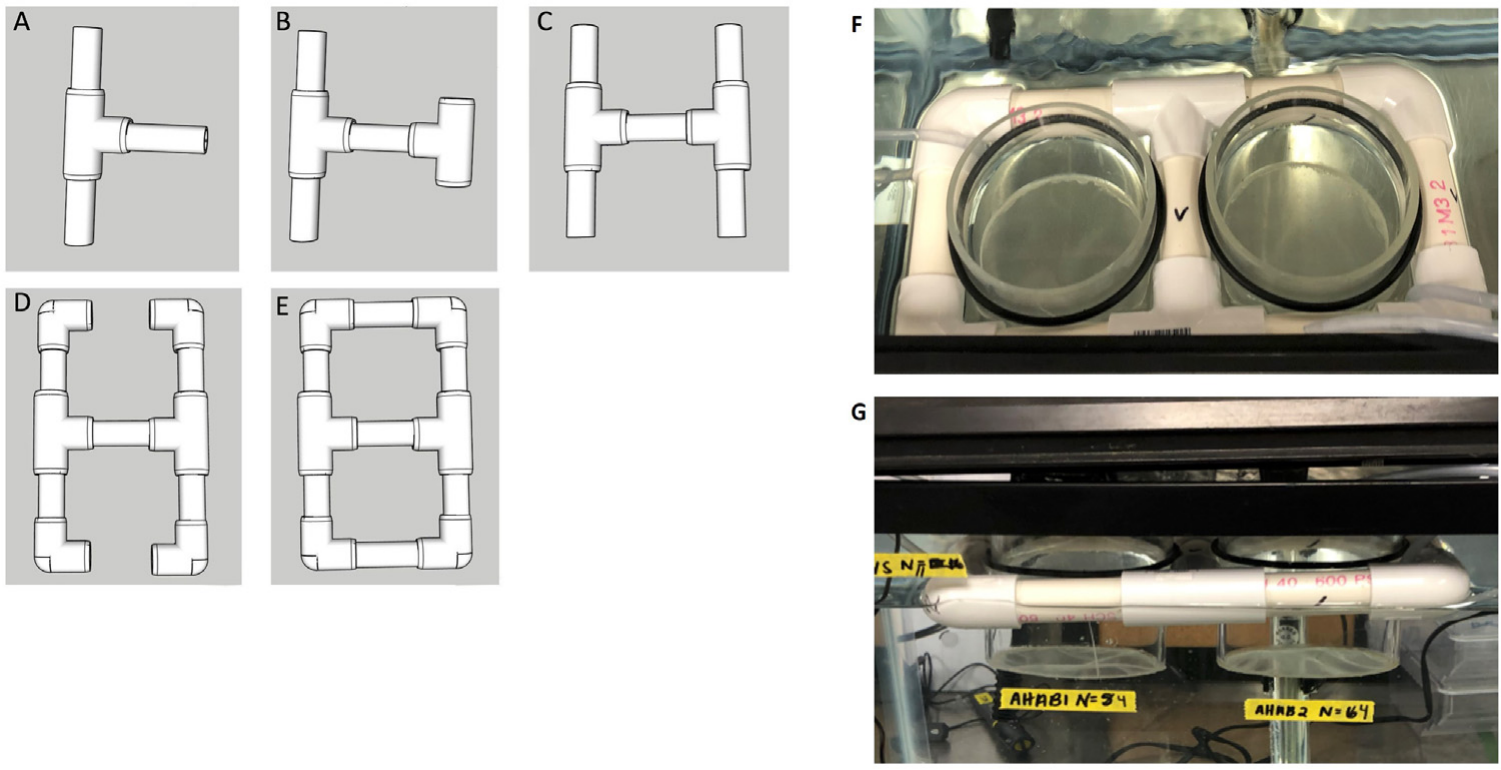
Assembly and configuration of FAST-MC. **(A)** PVC pieces are first inserted into a T-connector. **(B)** A second T-connector is added. **(C)** Additional PVC pieces are added to the second T-connector, forming an ‘H.’ **(D)** Corner pieces are added to the 4 open PVC ends. **(E)** The final PVC pieces are added, forming two openings. **(F)** The assembled PVC float with two mesh bottom containers floating in it. **(G)** The PVC float viewed from the side. The mesh bottom is suspended within an aquarium, providing continuous water flow and filtration.

*Assembly of FAST-MC:* The FAST-MC apparatus was composed of a standard zebrafish mesh bottom hatchery insert (Pentair, Apopka, FL) suspended in the 40-L aquarium by a homemade PVC float. PVC floats were constructed using 3″ (76 mm) long, ¾” (19 mm) diameter PVC piping that was cut to fit. Individual pipe pieces were connected by 90° elbow fittings (19 mm inner diameter) to form a square. This basic design can be modified to hold two hatcheries at the same time using PVC pipe T-connectors (Fig. 1). The fittings should be snug to prevent water from entering the pipe and to be the correct diameter to hold the hatchery. No PVC glue or other adhesive was used to keep the PVC apparatus together. All fittings were hand-tightened. Once assembled, the PVC float was placed within the aquarium to ensure it was watertight.

Next, the mesh-bottom insert was prepared. Our mesh-bottom inserts were the hatchery containers provided with the Aquatic Habitats unit. However, these mesh-bottom hatcheries can also be made by the user. Each hatchery was made of a clear acrylic cylinder (102 mm diameter, 115 mm tall) closed on one end by a piece of mesh nylon filter (400 µm mesh). The mesh was affixed to the cylinder with aquarium silicon and allowed to dry overnight. Around the outside of the hatchery cylinder, a black rubber ‘O’ ring was placed 13 mm (0.5″) from the open (non-mesh) side. Once the mesh and O-ring were in place, the hatchery was inserted into the PVC holder so the black ring rested on the PVC float. The PVC float + hatchery was the FAST-MC apparatus. Once assembled, the FAST-MC apparatus was placed into the 40-L aquarium (Fig. 1). Minor adjustments to the O-ring may be needed to secure the hatchery in the PVC float.

Once inserted, the FAST-MC was secured to the side of the aquarium using airline tubing. To do this, tubing was threaded through the corners of the apparatus (between the hatchery and the PVC float) on both sides and the ends connected to the side of the aquarium with a binder clip. This prevented the FAST-MC apparatus from moving around on the top of the water and drifting into the outflow from the top filter. To prevent evaporation, the aquarium was covered with the lid provided with the aquarium. Once assembled, the entire set-up remained undisturbed for one day to make sure everything was working properly.

### Use and cleaning of FAST-MC

Once the FAST-MC containers were assembled and floating in the aquarium, fish eggs and/or larvae were added to the individual containers. The water within the aquarium can be either system water or water that contains experimental chemicals. In this way, a single aquarium becomes an experimental treatment, and the individual FAST-MC hatcheries are the exposure/housing containers. This set up was analogous to having multiple petri dishes or multiple wells of a multi-well plate containing the same exposure treatment condition.

We found that the larger volume of the FAST-MC aquarium, and its attached filter, significantly reduced manual husbandry. Rather than the daily manual care required for petri dishes, the water within the aquarium was continually filtered through the installed top filter, which reduced/eliminated the need for manual water changes. Nonetheless, water quality measurements (temperature, pH, ammonia, nitrate, nitrite) were checked routinely within each aquarium and water was added weekly to compensate for evaporative loss.

Once the larvae were removed from the FAST-MC containers, the aquarium, filter, and FAST-MC apparatus were separated and cleaned following protocols within the fish facility. If system water was used (i.e., the eggs/larvae remain in the colony), all components were reused. If experimental water containing chemicals was used with the FAST-MC, experimenters may want to restrict usage to prevent contamination.

## Method validation

To validate our method, we raised zebrafish larvae for 4-weeks in either FAST-MC containers or deep dish/‘extra depth’ petri dishes (VWR; 26 mm depth; filled ½ to ⅔ full of system water) and compared water quality in the experimental containers as well as overall survival, general swimming behaviors (total distance traveled, angular velocity), and anxiety (open field test) of the larvae. We selected these parameters because they are well established and often reported in studies with zebrafish [18,19] and we hypothesized that they may be impacted by larval housing conditions. For both groups, viable fertilized eggs obtained from in-house spawning of adults [15] were cleaned and placed into the different housing conditions (both petri dishes and FAST-MC) on the same day of fertilization at a density of 6–10 eggs per container, with 2–4 containers per housing condition used for each spawn. Eggs from 4 different spawns were used for this study. Biological replicates were the individual housing containers (i.e., each petri dish or each FAST-MC container). Once within the petri dishes and FAST-MC containers, larvae were observed daily. Eggs/larvae within the petri dishes had daily water changes of 25–30 % to remove uneaten food, debris, and any dead larvae. Eggs/larvae in FAST-MC containers had water added to the aquarium approximately once a week to replace water lost to evaporation and any uneaten food that had fallen through the mesh bottom of the hatchery was removed at this time.

### Survival

**Fig. 2:**
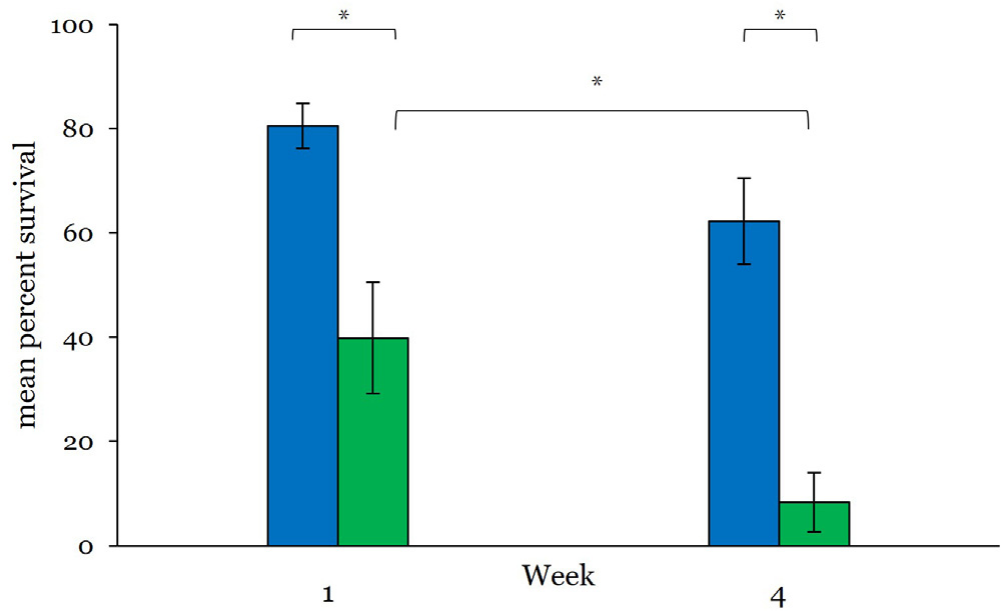
Survival. Survival of larval zebrafish differed by container, with greater overall survival noted in FAST-MC containers. After 1-week, survival in FAST-MS averaged 80 % compared to 40 % survival in the petri dishes (*p* < 0.001; *N* = 10 FAST-MC; *N* = 13 dishes). At 4-weeks, survival in FAST-MC containers (*N* = 7) was 60 % compared to an average 8 % survival in dishes (*N* = 10; *p* = 0.019). Overall survival was significantly different between weeks 1 and 4 for dish larvae only (*p* = 0.03). Asterisks denote significant differences Values are mean ± SE. Blue bars = FAST-MC; green bars = petri dishes.

Percent surviving in each housing condition was quantified after 1-week and 4-weeks. Overall, survival differed with type of housing container (Fig. 2). After 1-week in FAST-MC containers 81 ± 4.4 % of larvae survived (*N* = 10), which was significantly more than the 40 ± 10.7 % survival (*N* = 13) observed in petri dishes (*p* = 0.019; Mann-Whitney U (*n* = 23) = 27.5, *z* = -2.34). By week 4, survival within petri dishes was 8 ± 5.7 % (*N* = 10) which was significantly lower (*p* < 0.001; U (*n* = 17) = 2.5, *z* = -3.36) than the 62 ± 8.3 % survival in FAST-MC containers (*N* = 7). Overall survival was significantly different between weeks 1 and 4 for larvae within the dishes (*p* = 0.03, U (*n* = 23) = 30, *z* = -2.346). However, and importantly, survival was not found to be significantly different after 1-week vs. 4-weeks of housing in FAST-MC containers (*p* = 0.07, U (*n* = 17) = 16, *z* = -1.873).

### Water quality

Water quality measurements revealed that temperature, pH, and conductivity values were consistent within both the petri dishes and the FAST-MC containers across the 4 weeks (Table 1). However, ammonia, nitrite, and nitrate levels were higher in petri dishes. Ammonia levels increased in the petri dishes after 1-week, despite daily water changes, and remained elevated. Nitrite and nitrate levels were elevated at 2-weeks and increased an additional 3–4x by 4-weeks.

**Table 1:** Water quality parameters.

	Temperature	Conductivity	pH	nitrate	nitrite	ammonia
	77 (± 0.05)	1075 (± 42.5)	7.07 (± 0.23)	2.5 (± 1.44)	0.00	0.1 (± 0.07)
**Week 1**						
petri dish	73.20	Not	7.12 (± 0.07)	46.7 (± 12.29)	0.04 (± 0.04)	3.7 (± 1.38)
FAST-MC	76 (± 0.85)	measured	7.5 (± 0.06)	0.00	0.00	0.1 (± 0.06)
**Week 2**						
petri dish	75 (± 0.1)	1290 (± 89.2)	7.60	6.7 (± 6.67)	0.3 (± 0.33)	6.7 (± 1.33)
FAST-MC	77.00	1488 (± 158.5)	7.3 (± 0.3)	7.5 (± 2.5)	0.00	0.4 (± 0.13)
**Week 4**						
petri dish	75 (± 1)	1467 (± 33.5)	7.60	60 (± 20)	5.00	4.00
FAST-MC	76 (± 0.3)	1444.00	7.4 (± 0.2)	15 (± 5)	0.00	0.1 (± 0.13)

At weeks 1, 2, and 4 water quality was measured. Temperature (C), pH, and conductivity (µS/cm) were consistent across the time points in both types of containers. However, ammonia, nitrate, and nitrite (all in ppm) measurements were consistently larger in petri dishes compared to FAST-MC containers. Values are mean ± SE, if more than one measurement was collected at a given time point.

### Behavior

Based on the differences in water quality, fish behavior was recorded after 1-, 2-, and 4-weeks in each housing condition using Ethovision (version 15, Noldus Information Technology, Inc, Leesburg, VA). Recordings were made using a 6-well plate, with 1 larva per well. Once in the well plate, fish were given 5 min to acclimate to the recording chamber before the 5 min recording began. Each well was filled ½ to ⅔ full of system water and fish were tested individually. Total distance moved and angular velocity measurements were determined for each larva for the entire 5 min recording period. To measure anxiety in an open field, we assessed the position of each larva within the ‘center’ or ‘edge’ of the well during the 5 min recording period. The ‘center’ was defined as the middle 50 % of the well and the ‘edge’ defined as the outer 50 % around the center. For all behaviors, EthoVision tracked each individual larva and measured swimming movements and location within the recording dish in real time. For analysis, the cumulative total distance (mm), angular velocity (deg/s), and position measurements collected from each larva during the entire 5 min recording period were used. As each container was a replicate, the values obtained from all larvae within a given container were averaged to provide a single value per container for each parameter for statistical analysis. Outliers were identified using Dixon’s text and removed before evaluating differences due to housing condition at each time point with a Mann Whitney test. All statistical tests were performed in SPSS (IBM) and evaluated at α = 0.05. Graphs were made in Excel.

**Fig. 3:**
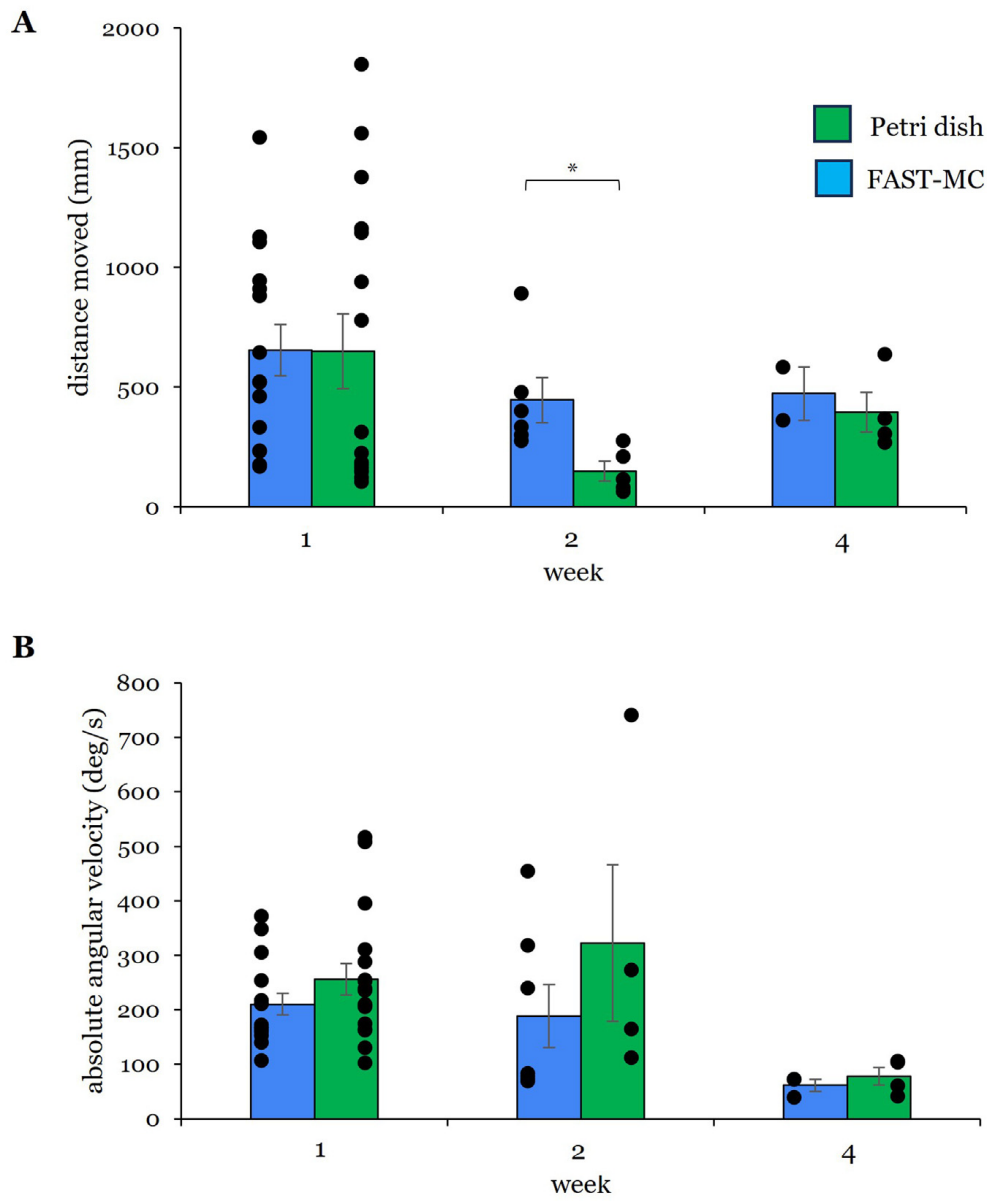
Swimming behaviors. **(A)** Total distance moved (mm) and **(B)** absolute angular velocity (deg/*sec*) were calculated after 1-week, 2-weeks, and 4-weeks. Larvae raised in FAST-MC **(A)** moved a significantly greater average distance than larvae raised in petri dishes at 2-weeks (*p* = 0.009). Week 1: *N* = 15 FAST-MC; *N* = 16 dish | Week 2: *N* = 6 FAST-MC; *N* = 5 dish | Week 4: *N* = 2 FAST-MC; *N* = 4 dish) **(B)** No differences were observed in mean absolute angular velocity measurements at any time point, though FAST-MC larvae had consistently lower values at all time points. Week 1: *N* = 15 FAST-MC; *N* = 17 dish | Week 2: *N* = 7 FAST-MC; *N* = 4 dish | Week 4: *N* = 3 FAST-MC; *N* = 4 dish. Bar graphs show mean ± SE. Significance is indicated by asterisks. Blue = FAST-MC; green = petri dishes. Dots are individual data points from biological replicates.

Total distance moved (Fig. 3A) differed significantly due to housing condition at 2-weeks. After 1-week, zebrafish larvae housed in FAST-MC containers moved an average of 654 ± 107 mm (mean ± SE, *N* = 15), which was similar to the average distance traveled by larvae raised in petri dishes (650 ± 155.7 mm, *N* = 16). However, after 2-weeks of housing in FAST-MC, larvae moved significantly farther (*p* = 0.009, U (*n* = 11) = 29, *z* = 2.556) than larvae in the petri dishes (FAST-MC: 447 ± 94 mm, *N* = 6 | dish: 149 ± 41 mm, *N* = 5). Examining the individual data points for the same data (Fig. 3A) shows greater overall movement at 1-week for both groups of larvae and greater movement of FAST-MC larvae at 2-weeks. Absolute angular velocity (Fig. 3B) was not significantly altered due to housing container, though values were lower for FAST-MC larvae at all three time points. Examination of individual data points revealed large variability in angular velocity measurements at the 1- and 2-week time points.

**Fig. 4:**
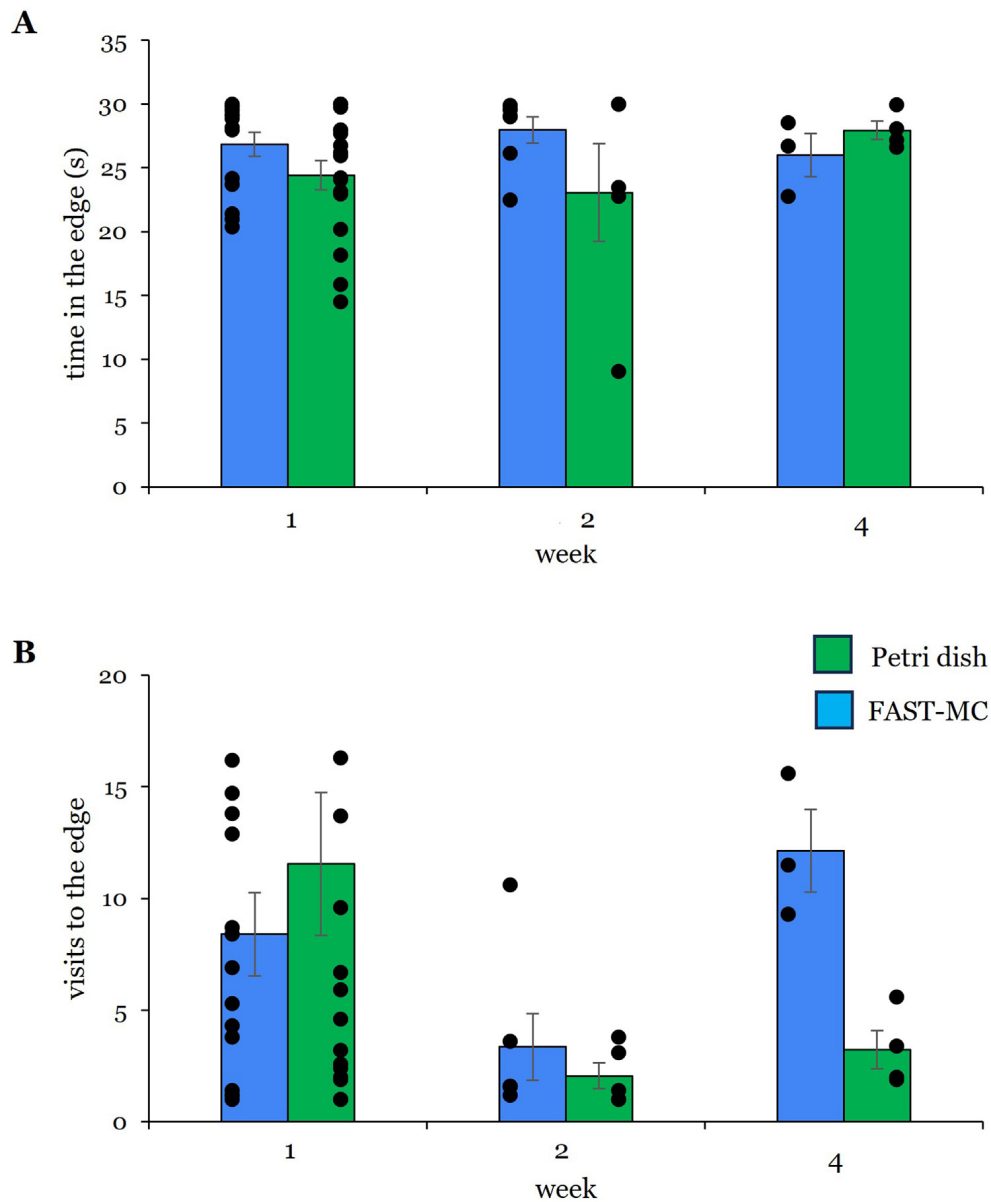
Time and visits to the edge. Position at the edge of the dish is a measure of thigmotaxis in zebrafish larvae. **(A)** No difference in time in at the edge of the dish was observed Week 1: *N* = 15 FAST-MC; *N* = 17 dish | Week 2: *N* = 7 FAST-MC; *N* = 5 dish | Week 4: *N* = 3 FAST-MC; *N* = 4 dish. **(B)** Though larvae housed in FAST-MC made 3x more visits to the edge at 4-weeks, this difference was only marginally not significant (*p* = 0.057). Individual data points suggest the most visits to the edge were made by all larvae at 1-week. Week 1: *N* = 15 FAST-MC; *N* = 17 dish | Week 2: *N* = 6 FAST-MC; *N* = 5 dish | Week 4: *N* = 3 FAST-MC; *N* = 4 dish. Bar graphs show mean ± SE. Blue bars = FAST-MC; green bars = petri dish. Dots are individual data points from biological replicates.

Within the open field, larvae housed in the FAST-MC containers averaged 27 ± 0.94 s (*N* = 15) at the edge of the dish after 1-week (Fig. 4A), which was not significantly different from the time spent at the edge of the dish by larvae housed in petri dishes (24 ± 1.17 s, *N* = 17) (*p* = 0.123, U (*n* = 32) = 169, *z* = 1.568). FAST-MC larvae also spent more time at the edge at 2-weeks (FAST-MC: 28 ± 0.1.03 s, *N* = 7 |petri dishes: 23 ± 3.8 s, *N* = 5), but these values were not significantly different (*p* = 0.876, U (*n* = 12) = 19, *z* = 0.244) However, examination of the individual data points suggests that, over the entire 4-week period, larvae housed in petri dishes spent more time at the edge at 1-week and 2-weeks overall, whereas larvae housed in FAST-MC containers spent approximately the same time at the edge across the 3 time points (Fig. 4A). The number of visits to the edge (Fig. 4B) made by FAST-MC larvae at 1-week was less than the number of visits by petri dish larvae. At 4-weeks, however, FAST-MC larvae made an average of 12 ± 0.1.8 visits, compared to an average of 3 ± 0.86 visits, a trending difference (*p* = 0.057, U (*n* = 7) = 12, *z* = 2.121).

**Fig. 5:**
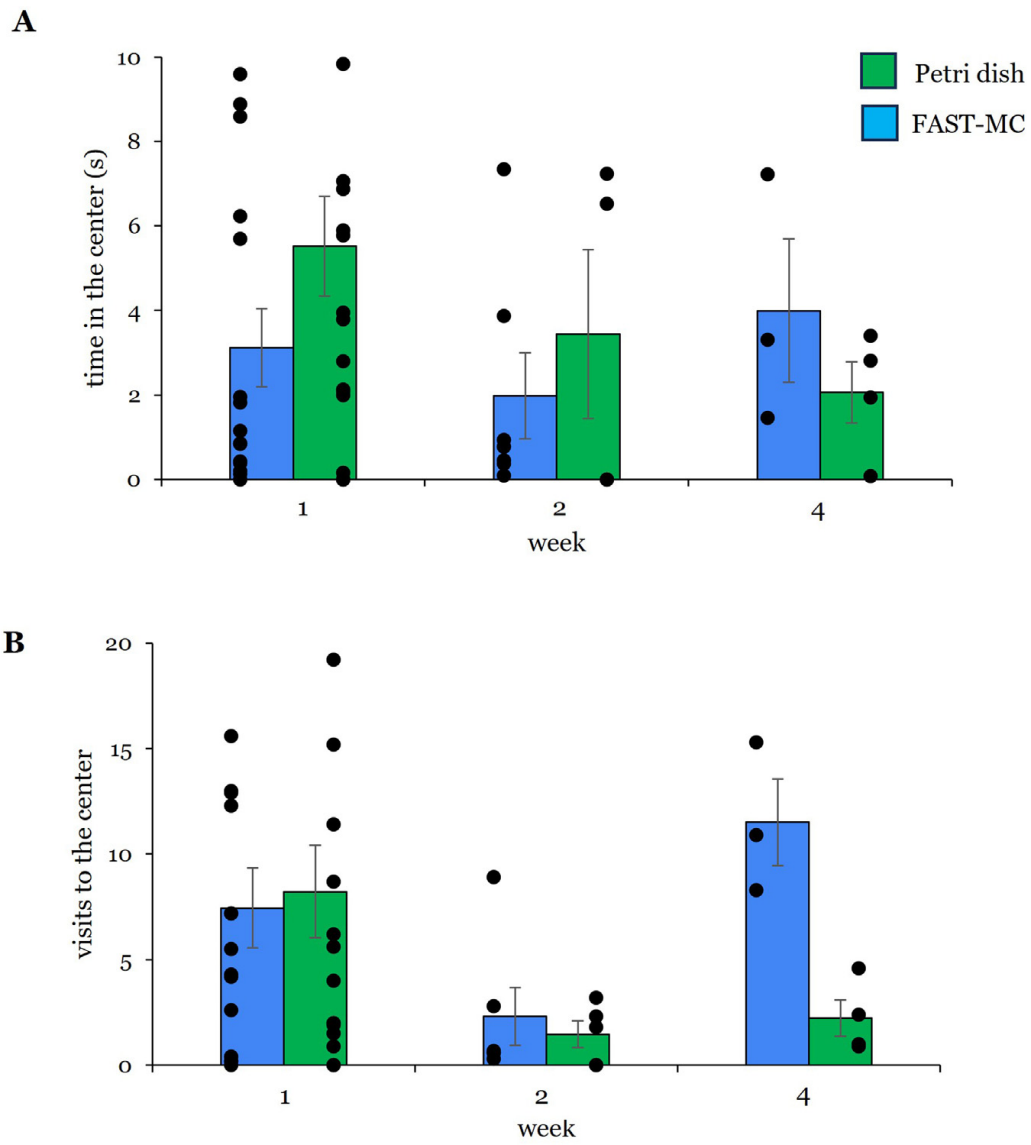
Time and visits to the center. **(A)** No difference was observed in the amount of time larvae spent in the center of the dish and individual data points show high overall variability in responses for both petri dish and FAST-MC larvae at all time points. Week 1: *N* = 15 FAST-MC; *N* = 17 dish | Week 2: *N* = 7 FAST-MC; *N* = 4 dish | Week 4: *N* = 3 FAST-MC; *N* = 4 dish. **(B)** FAST-MC larvae the most visits to the center at 4-weeks, though this value was not significant (*p* = 0.057) compared to larvae housed in petri dishes. No other differences in visits to the center were observed, though the greatest movement occurred at 1-week. Week 1: *N* = 15 FAST-MC; *N* = 17 dish | Week 2: *N* = 6 FAST-MC, *N* = 5 dish | Week 4: *N* = 3 FAST-MC, *N* = 4 dish. Bar graphs are mean ± SE. Blue = FAST-MC; green = petri dishes. Dots are individual data points from biological replicates.

The amount of time FAST-MC larvae spent in the center of the recording dish (Fig. 5A) was less than petri dish larvae at 1-weeks (3 ± 0.93 s FAST-MC | 6 ± 1.2 s petri dish) and 2-weeks (2 ± 1.02 s FAST-MC | 3 ± 1.99 s dish). At 4-weeks, however, FAST-MC larvae spent 2x more time in the center than larvae raised in petri dishes (4 ± 1.7 s FAST-MC | 2 ± 0.73 s dish). FAST-MC larvae made fewer visits to the center of the dish than petri dish larvae at 1-week (7 ± 1.9 FAST-MC | 8 ± 2.2 dish); while more visits to the center were made by FAST-MC larvae at weeks 2 and 4. The most visits to the center by FAST-MC larvae occurred at 4-weeks (12 ± 2) compared to larvae raised in petri dishes (2 ± 0.86). Statistically, the 6x difference in center visits observed at 4-weeks was not significant (*p* = 0.057, U (*n* = 7) = 12, *z* = 2.121). Overall, larvae housed in petri dishes visited the center the most at 1-week (Fig. 5B individual data points); while larvae raised in FAST-MC containers appeared to visit the center more consistently across all 4 weeks.

Thus, our validation experiments show that the FAST-MC method provides an alternate option for larval zebrafish housing compared to more traditional petri dishes (i.e., [15]). We observed better survival and consistent water quality which were associated with increased overall activity by larvae housed within the FAST-MC containers. Our method is similar to the MaxHatch ™ nursery described by Cattin and Crosier [17]. They also used a flow through system to maintain water quality and showed improved survival vs. more conventional methods. However, their system is large. The smaller aquaria used with FAST-MC containers would be useful for smaller labs and/or toxicological studies that require larval exposures to last for several weeks.

Larvae raised in either housing condition were most active (i.e., displayed the greatest data range) at 1-week. At 2-weeks and 4-weeks, this variability was reduced, though activity tended to be higher overall for larvae raised in FAST-MC containers. The increased activity of zebrafish larvae in FAST-MC housing containers suggests purposeful movement. These larvae showed a consistent decrease in average angular velocity across all 4 weeks, the mean the total distance traveled increased compared to larvae housed in petri dishes at weeks 2 and . This steady/increasing distance moved with fewer changes in direction suggests purposeful movement [20] by FAST-MC fish. In contrast, larvae maintained in petri dishes had inconsistent angular velocity and total distance moved measurements across assessment times. Their average total distance traveled decreased from 1-week to 2-weeks but then increased between 2-weeks and 4-weeks, suggesting little purposeful movement, as may occur if the fish made circular movements/remained in one place. These findings are reminiscent of ‘stereotypical behaviors’ reported for adult zebrafish housed in small vs. large tanks [7]. While the decrease in overall responses from larvae housed in petri dishes likely reflects the reduced water quality and water renewal rates observed in these containers, the results with adult zebrafish suggest that the overall depth (size) of the housing container may also play a role. Zebrafish adults housed in smaller tanks were observed to have lower stamina and decreased boldness compared to adults housed in larger tanks [7]. Water depth in FAST-MC containers was 51–64 mm in compared to < 26 mm in the petri dishes, making FAST-MC containers ∼2x larger in volume than the petri dishes. This difference may contribute to the activity differences noted between larvae.

Further evidence of increased activity by FAST-MC fish is seen in the open field test results. Larvae housed in either FAST-MC containers or petri dishes spent more time overall at the edge (∼25–30 s) of the recording dish than in the center (∼5 s), suggesting a similar level of anxiety [[21], [22], [23]]. However, FAST-MC larvae spent more time at the edge, particularly at the early (1-week and 2-week) time points. On the surface, this suggests that larvae housed in FAST-MC containers had increased anxiety, possibly due to the presence of the top filter in the aquarium. Alternatively, it could reflect movement from a deep/larger housing environment to a smaller recording chamber. Recordings were made in 6-well plates. Though all larvae were allowed to acclimate in the recording chamber for 5 min, larvae housed in petri dishes moved from one shallow dish to another shallow dish, whereas FAST-MC larvae moved from a deep container to a shallow dish. It’s typical to see more visits to either the center or the edge, but larvae housed in FAST-MC containers made more visits to the center and to the edge for the last weeks of the exposure. This increased movement between the center and edge of the recording chamber also supports increased movement and we conclude that spending more time in the edge, when paired with swimming in and out of the center and edge, suggests exploratory behavior in a new environment, as found in bold adult zebrafish [19] and in other study with zebrafish larvae using the open field test [24].

We hypothesized that water quality differences within the two housing conditions may correlate with the observed differences in survival and behavior. In a recirculating aquaculture system, where adult fish are typically housed, water renewal is frequent and nitrification or biological filtration occurs with the presence of beneficial bacteria [25]. This latter process converts the ammonia excreted by the fish [25] into nitrite, which is then converted to nitrate [25,26]. Nitrate, which cannot be broken down by bacteria or filtration, is removed by a water change [25]. As Petri dishes are static environments without filtration, the only way to manage water quality parameters is to perform daily water changes to remove debris and food waste. The FAST-MC containers, however, are submerged in aquarium water processed through a biological top filter, resulting in higher water renewal rates compared to petri dishes.

Because of their toxicity, ammonia and nitrite levels of 0 ppm are the ideal environment for fish [25,26]. Within the lab, an acceptable range for ammonia is 0.0–0.10 mg/L (ppm) [27]; another study recommends <1 ppm of total ammonia [25]. We observed ammonia levels of 0.1–0.4 ppm in FAST-MC containers, which contrast values of 4–7 ppm in the petri dishes. High levels of these nitrogen-containing compounds are deleterious to larvae [16] and likely contributed to the increased mortality in the petri dishes {Goodwin, 2016 #10;Hammer, 2020 #23}. However, it does appear that nitrification was occurring in petri dish water samples, as high ammonia levels were observed at 2-weeks, but high nitrate and nitrite levels were seen at 4-weeks. Nitrite levels in the FAST-MC containers were 0 ppm throughout the study. Recommended nitrate levels range from 50 ppm [25] to < 200 ppm [16,26]. In our samples, nitrate levels were lowest after 1-week and then increased with time, displaying the greatest value (15 ppm) after 4-weeks. While the overall values were within safe limits, the values within the petri dishes were much larger than the values in the FAST-MC containers, particularly at weeks 1 and 4.

Together, the above data identifies FAST-MC as an alternative method for housing larval zebrafish that allows for decreased mortality, improved water quality, and less time spent on animal care. The findings presented here demonstrate that larvae housed in FAST-MC containers for 4-weeks exhibited increased activity and purposeful movement compared to larvae housed in petri dishes for the same duration. The FAST-MC method would be an excellent option for research where exposures are greater than one week and may be especially useful in ecotoxicology.

### Limitations

The FAST-MC method provides an alternative option for larval zebrafish housing that decreased mortality and resulted in less time spent on animal care, which is optimal for long-term experimental exposures. Water quality testing found that FAST-MC containers had water quality parameters similar to system water with far less maintenance than petri dishes. Despite these positives, there are some limitations to the technique.

First, experimenters would need to dedicate at least one 40-L aquarium to a given exposure or breeding group. These aquaria are much larger and take up more counter space than petri dishes. Several petri dishes can be stacked within an incubator, allowing several dishes and treatments to be maintained at the same time. Thus, one limitation of FAST-MC is having enough counter space to run separate treatments simultaneously.

Second, we acknowledge that the larger volume of the aquaria could potentially necessitate more reagents, which would be more costly compared to the reduced volume in well plates or dishes. However, we feel this is offset by the better overall water quality and improved survival in FAST-MC containers. The FAST-MC housing system would also be useful for breeding zebrafish to maintain lines in the colony, and for separating offspring phenotypes from crosses using transgenic and/or mutant lines.

Third, if there was a deleterious situation in the aquarium, it would affect all FAST-MC containers. This is a more serious situation than would occur if one petri dish out of many had a problem. However, we feel that this is balanced by easier husbandry and maintenance when using FAST-MC. We also note that the 25–30 % daily water change daily in petri dishes is lower than what is reported [28] and may also contribute to the differences in water quality. Future studies should compare the amount of water change in the dishes with FAST-MC to address this concern.

Finally, when assembling the PVC floats, hand tightening may not make the float watertight. If this is the case, PVC glue or another sealant may need to be used. If this is the case, once dried, the glued PVC float should be soaked and/or washed several times thoroughly to leach out any potentially harmful chemicals.

## Ethics statements

Wild-type zebrafish (*Danio rerio*) were spawned in-house. Breeding fish were placed in tanks with a 4:1 female-to-male ratio in the afternoon, and fertilized eggs were collected next day. Zebrafish were maintained at 28±3 °C on a 14:10 hr light-dark cycle. Larvae were fed AP100 starting at 3 days postfertilization (dpf) [29] and at 7 dpf, live brine shrimp were introduced as a food supplement. The food regime, quantity of food, and timing of feeding was the same for both petri dishes and FAST-MC containers. All animal procedures were approved by AU’s Institutional Animal Care and Use Committee and in compliance with the ARRIVE guidelines and the NIH Guide for the Care and Use of Laboratory Animals.

## CRediT authorship contribution statement

**M. Caballero:** Conceptualization, Methodology, Formal analysis, Writing – original draft, Writing – review & editing. **S. Robles:** Methodology. **VP Connaughton:** Conceptualization, Funding acquisition, Formal analysis, Project administration, Supervision, Writing – review & editing.

## Declaration of competing interest

The authors declare that they have no known competing financial interests or personal relationships that could have appeared to influence the work reported in this paper.

## Data Availability

Data will be made available on request.
